# Suicide in young adults: psychiatric and socio-economic factors from a case–control study

**DOI:** 10.1186/1471-244X-14-68

**Published:** 2014-03-06

**Authors:** Andrew Page, Stephen Morrell, Coletta Hobbs, Greg Carter, Michael Dudley, Johan Duflou, Richard Taylor

**Affiliations:** 1 School of Science and Health, University of Western Sydney, Campbelltown Campus, Locked Bag 1797, Penrith, NSW 2571, Australia; 2 School of Public Health and Community Medicine, University of New South Wales, Samuels Building, Botany Street, Randwick, NSW 2052, Australia; 3 Sydney School of Public Health, University of Sydney, Edward Ford Building, Camperdown, NSW 2006, Australia; 4 Centre for Translational Neuroscience and Mental Health (CTNMH), University of Newcastle, Locked Bag 7, Hunter Region Mail Centre, Newcastle, New South Wales NSW 2310, Australia; 5 Department of Consultation-Liaison Psychiatry, Calvary Mater Newcastle Hospital, Locked Bag 7, Hunter Region Mail Centre, Newcastle, New South Wales NSW 2310, Australia; 6 School of Psychiatry, University of New South Wales, Hospital Road, Prince of Wales Hospital, Randwick NSW 2031, Australia; 7 Sydney Medical School, University of Sydney, Sydney, NSW 2006, Australia; 8 Department of Forensic Medicine, New South Wales Health Pathology, PO Box 90, Glebe, NSW 2037, Australia

**Keywords:** Suicide, Socio-economic factors, Anxiety disorders, Depressive disorders, Substance use disorders, Population attributable risk

## Abstract

**Background:**

Suicide in young adults remains an important public health issue in Australia. The attributable risks associated with broader socioeconomic factors, compared to more proximal psychiatric disorders, have not been considered previously in individual-level studies of young adults. This study compared the relative contributions of psychiatric disorder and socio-economic disadvantage associated with suicide in terms of relative and attributable risk in young adults.

**Method:**

A population-based case–control study of young adults (18–34 years) compared cases of suicide (n = 84) with randomly selected controls (n = 250) from population catchments in New South Wales (Australia), with exposure information collected from key informant interviews (for both cases and controls). The relative and attributable risk of suicide associated with ICD-10 defined substance use, affective, and anxiety disorder was compared with educational achievement and household income, adjusting for key confounders. Prevalence of exposures from the control group was used to estimate population attributable fractions (PAF).

**Results:**

Strong associations were evident between mental disorders and suicide for both males and females (ORs 3.1 to 18.7). The strongest association was for anxiety disorders (both males and females), followed by affective disorders and substance use disorders. Associations for socio-economic status were smaller in magnitude than for mental disorders for both males and females (ORs 1.1 to 4.8 for lower compared to high SES groups). The combined PAF% for all mental disorders (48% for males and 52% for females) was similar in magnitude to socio-economic status (46% for males and 58% for females).

**Conclusion:**

Socio-economic status had a similar magnitude of population attributable risk for suicide as mental disorders. Public health interventions to reduce suicide should incorporate socio-economic disadvantage in addition to mental illness as a potential target for intervention.

## Background

Suicide remains an important public health problem in Australia, particularly in young adult males (aged 18–34 years) [1,2]. Like many conditions and health outcomes in public health, the aetiology of young adult suicide is complex and multidimensional, involving a range of distal, intermediate and proximal causes [3].

Previous individual-level, population-based studies have shown an increased risk of suicide associated with psychiatric disorders, especially affective disorders [4-6], substance use disorders [5,7,8], and schizophrenia [5,9]. The relative risk of suicide associated with these proximal risk factors is high, with relative risk (RR) estimates for those with (compared to those without) a mental disorder ranging from RR ≈ 2 to RR ≈ 14 [10]. However, psychiatric disorders, are relatively uncommon in the general population with median period prevalence ranging from approximately 1% to 12% based on control groups in individual-level, population-based studies [10]. Such prevalences are consistent with 12-month population prevalences found in Australian mental health surveys, ranging from 5% to 14% [11]. These mental disorders have available partially effective treatments and prevention interventions, and so are generally accepted as legitimate targets for a public health approach to suicide prevention.

More distal risk factors associated with suicide, such as measures of socio-economic status, have been shown to have substantially lower relative risk estimates associated with suicide in population-based studies (RR for low versus high SES groups ranging from 1.3 to 2.7) [10]. However, the proportion of the population exposed to low socio-economic status is much greater than the prevalence of psychiatric disorders. Such distal risk factors, with greater population exposure have not traditionally been the focus of suicide prevention targets in public health approaches or in national suicide prevention policies.

The overall importance of a risk factor for suicide in a population is a combination of both the relative and absolute risk. Population attributable risk is a measure that combines both of these components in a population, and can be used to compare the relative importance in terms of public health importance and disease burden [12,13], and can be used in the identification and prioritisation of suitable targets for public health interventions. A recent systematic review of individual-level, population-based studies of adult suicide showed that the population attributable risk of some measures of socio-economic status (particularly low educational achievement) was of a similar magnitude to psychiatric disorders (including substance use and anxiety disorders) [10].

The relative contribution of socio-economic and psychiatric factors to attempted suicide has previously been shown to be similar in Australia in community-based [14,15] and clinical populations [14], although these studies combined 12-month and lifetime prevalence measures of mental disorder in analyses. A number of individual-level population-based case–control studies have been conducted internationally [4,6-8,16-18] including both socio-economic and measures of mental disorder based on ICD/DSM diagnosis, however, these studies have usually focussed on adolescents [6,7,16], a single gender [17] or older-age groups [4,8,18]. Previous studies have not investigated the relative contribution of socio-economic and psychiatric factors using individual-level population-based data, with the exception of the large nested case–control studies from the Danish record linkage studies [5,19] however, these latter studies did not focus on young adults, an age-group where suicide rates have been highest since the 1980s in industrialised countries [20]. The present study uses data collected from a recent population-based case–control interview study of young adults in New South Wales (Australia) to investigate: the association between psychiatric disorders and suicide; the association between key socio-economic measures and suicide; and the population attributable risk estimates of both socio-economic and psychiatric factors.

## Methods

### Study design

This is a population-based case–control study of suicide in young adults (18–34 years) in defined population catchments in New South Wales (NSW), Australia. Information on suicide cases and controls was based on third-person (next of kin) information obtained from face-to-face interviews relating to indexed cases and controls. The study received institutional ethical approval from jurisdictions relating to the relevant coronial, and community-based catchments of the research, including the University of Sydney, Southern Sydney Area Health Service (AHS), Greater Western AHS, Western Sydney AHS, Hunter New England AHS, South Eastern Sydney AHS (Eastern), South Eastern Sydney AHS (Southern), The National Coronial Information System (NCIS), Department of Justice Victoria, and the NSW Aboriginal Medical Health and Research Council Ethics Committee.

### Suicide cases

The National Coronial Information System (NCIS) was accessed to identify all 18–34 year old male and female suicide cases in the Coronial Court jurisdictions of Sydney, Westmead, and Newcastle (urban), and Maitland, East Maitland, Bathurst, Orange and Dubbo (rural), in NSW for the period 2003–08. The definition of a suicide case was based on the coronial determination of intentional self-harm, supplemented by pathology report summaries. A Coronial file audit was conducted for each case to enumerate contact details for the next-of–kin or ‘significant others’, and an invitation to participate in the study was sent to potential study participants on behalf of the NSW Chief Forensic Pathologist. Of the 219 suicides in the coronial data extracted by the authors, the next-of-kin could be contacted using the available contact information in 120 cases. Consenting next-of-kin of suicide cases (n = 84, 70% response) participated in face-to-face interviews to complete a questionnaire about socio-demographic factors, life events and other antecedent circumstances of the suicide case.

### Controls

The Australian Bureau of Statistics (ABS) provided a sampling frame derived from the Census of Population and Housing and the Monthly Population Survey (MPS) design framework. The Socio-Economic Index of Economic Resources (SEIFA) [21] was then applied to the frame, with Collection Districts (CDs) selected systematically according to the SEIFA characteristics which would represent the Local Government Area (LGA) characteristics covered by the coronial and hospital jurisdictions described above. These processes provided selected CDs for control households within the LGA areas, where there was an 80% probability that the residence contained young adults for strata matching to the sex and age group (within 2–3 years) of the cases. No other characteristics apart from age and sex were matched for in analyses. Potential controls were located via door-knocking in these catchment areas using CD maps provided by the ABS.

Controls (n = 250) were those aged 18–34 years and who resided in the regions from which suicide cases were drawn. Of the 1,439 households approached, 304 relatives or friends were nominated, from which a total of 250 interviews were conducted (response 82%). More controls than cases were recruited (3:1 control to case ratio for the total sample) to maximise statistical power given the small number of cases in the geographic catchments that could be accrued over the study period. Suicide controls were asked to nominate an informant (parent, relative, or friend), and the informant completed the same interview as completed by the next of kin of suicide cases. As for cases, the interview for suicide controls was conducted in a face-to-face format, and related to socio-demographic factors, life-events and other suicide risk factors. The use of third person information on an index suicide control enabled comparison with third person information collected on suicide cases, and ensured that a likely source of recall bias in psychological autopsy studies was more consistent between cases and controls than designs using first-person interviews of live controls, and minimised the effects of recall bias on relative differences in exposures between cases and controls.

*Post hoc* analysis of recruited controls for metropolitan Sydney and Hunter Region catchments showed similar sex- and age-distributions to the 2001 Census for corresponding areas, although a higher proportion of 20–24 year females were recruited, and a lower proportion of 30–34 year males were recruited than in the population. The proportion of males aged 20–24, 25–29 and 30–34 in the Census population was 14%, 15%, and 16%, and for females 14%, 16% and 16%. Corresponding proportions for male suicide controls were 12%, 13%, 9% and for female suicide controls were 22%, 14%, 13%.

### Survey data collection

An electronic interview questionnaire was developed specifically for this study to enable collection of data by laptop computer during the interview. The structured interview questionnaire was derived from standard psychiatric and psychological instruments, the National Survey of Mental Health and Well-being (NSMHWB) and standard population surveys (particularly the Australian Health Survey) and the Australian Census. Cases and controls were interviewed by trained clinical interviewers with health, medical or psychology qualifications to minimise interviewer and recall bias. Interviewers were not blinded as to whether the interview related to a case or control, given the sensitive nature of the content of the interview, and the need to have differing information and consent processes for the interview schedules for next-of-kin of suicide cases compared to next-of-kin for suicide controls. The interview questions for the present study focused on the following domains: (1) socio-demographic factors, including income, education, occupation, employment status, and marital status, among others; (2) family and childhood factors, including individual and family history of mental health illness and psychiatric symptoms; (3) psychiatric disorders, including affective disorders, alcohol and substance use disorders, personality disorders, and psychosis.

### Psychiatric disorder

The questionnaire of the World Health Organization Composite International Diagnostic Interview (CIDI) was used in the study for collecting self-report symptoms, which were used to score ICD-10 mental disorder diagnosis [22-24]. High prevalence mental disorders were the focus of the present study, and included substance use disorders (F10-F19), affective disorders (F30-F34), and anxiety disorders (F40-F43).

### Socio-economic status

A composite income and education level variable was derived as a measure of socio-economic status for analyses. Educational achievement was categorised as ‘no secondary school qualification’, ‘secondary school qualification’, ‘Trade certificate or diploma’, or ‘university degree or higher’. Household income level was categorised ‘$0-$29,999’, ‘$30,000-$69,999’, and ‘>$70,000’. The composite measure was calculated by addition of the categorical education and income level scores, resulting in a 5-level socio-economic status variable. Based on the distribution of study participants and the similar effect sizes between categories, the lower two categories were grouped together, as were the upper two categories, resulting in a composite SES measure representing ‘low’, ‘middle’, and ‘high’ SES groups. This composite SES measure based on education level and household income was adopted as preliminary analyses suggested that differences in these components of SES showed the strongest association with suicide. Due to insufficient case numbers and missing values in occupational and employment categories, occupational and employment could not be analysed individually with regard to suicide.

### Other factors

Marital status and family history of mental disorder were also included as confounding factors. These variables showed strong associations in preliminary univariable analyses and were specified as potential common causes (confounders) of the associations between SES, psychiatric disorder and suicidal behaviour. Marital status was defined as ‘married or *de facto*’, ‘separated, divorced, or widowed’, or ‘never married’. A family history of mental health problems was also included as a proxy measure of a potential predisposition to mental disorder which may be associated with both current socio-economic status and mental disorder. Participants were asked whether the case or control had a mother, father or grandparent with a history of depression, a drug or alcohol use problem, or an anxiety disorder.

### Analysis

The distribution of cases and controls, stratified by sex, were investigated for socio-economic status variables, psychiatric disorder, and potential confounders in a series of univariable analyses. Conditional logistic regression models, matched on age-strata and stratified by sex, were conducted to estimate the relative risk of socio-economic status variables and psychiatric disorders. Analyses were stratified by sex *a priori* as the epidemiology of suicide is different in males compared to females in Australia, where the incidence is approximately 4 times higher in males than females [2], and also allows comparability of sex-specific relative risk estimates with other population-based case–control and cohort studies [10]. Multivariable models were also conducted to adjust for potential confounders. Regression modelling was conducted in Stata Version 12.1 [25] using the *clogit* function. Adjusted population attributable fractions (PAF%) for lower socio-economic status (low and middle SES compared to high SES) and psychiatric disorder variables were obtained from the *punafcc* post-estimation function in Stata [25] based on the method used by Greenland and Drescher [26]. PAF% estimates for each exposure are not additive, given that individuals may experience multiple exposures [27]. A combined PAF for ‘any mental disorder’ (substance use, affective, or anxiety disorders) was also calculated using the *punafcc* post-estimation function.

## Results

There were 71 male and 13 female suicide cases, along with 102 male and 142 female age-matched controls, with higher risk of suicide in males compared to females (OR = 7.46, 95%CI 3.89-14.32, not shown). However, there was no statistical evidence of effect modification by sex for each of the main study factors (*P* value range 0.3778-0.833) due to small numbers of suicide cases, especially in females (Table 1). Strong associations were evident between mental disorders and suicide for both males and females in univariable analyses ranging from OR = 3.47 (95%CI 1.71-7.03, *P* < 0.001) for substance use disorder in males to OR = 18.68 (95%CI3.13-111.34) for anxiety disorders in females (Table 1), which were attenuated following adjustment for socio-economic status and other variables (Table 2). In males, anxiety disorders were most strongly associated with suicide (OR = 5.25, 95%CI 1.18-23.45, *P* = 0.030), followed by Affective Disorders (OR = 3.63, 95%CI 1.48-8.91, *P* = 0.005), and Substance Use Disorder (OR = 3.26, 95%CI 1.45-7.36, *P* = 0.004), after adjusting for marital status, family history and socio-economic status (Table 2). Similarly, for female suicide, the strongest association was for anxiety disorders (OR = 67.93, 95%CI 14.03-328.79, *P* = 0.008); the relatively small number of female suicide cases is reflected in the wide confidence interval (Table 2).

**Table 1:** Suicide in young adults aged 18–34 years: distribution and bivariate associations with study factors (84 cases, 250 controls) 2003–2008, New South Wales (Australia)

	**Males**						**Females**					
	**Cases**		**Controls**				**Cases**		**Controls**			
	**%**	n/N*	**%**	n/N*	**OR (95%CI)**	**P value**	**%**	n/N*	**%**	n/N*	**OR (95%CI)**	**P value**
*ICD mental disorder*												
Substance use disorder												
No	54.9	39/71	82.4	84/102	1.00		69.2	9/13	90.5	134/148	1.00	
Yes	45.1	32/71	17.6	18/102	3.47 (1.71-7.03)	<0.001	30.8	4/13	9.5	14/148	4.26 (1.12-16.24)	0.034
Affective disorder	.						.					
No	60.6	43/71	88.2	90/102	1.00		61.5	8/13	89.2	132/148	1.00	
Yes	39.4	28/71	11.8	12/102	4.26 (1.95-9.27)	<0.001	38.5	5/13	10.8	16/148	4.97 (1.45-17.05)	0.011
Anxiety disorder	.						.					
No	84.5	60/71	97.1	99/102	1.00		69.2	9/13	95.9	142/148	1.00	
Yes	15.5	11/71	2.9	3/102	5.45 (1.38-21.47)	0.015	30.8	4/13	4.1	6/148	18.68 (3.13-111.34)	0.001
*Family history of mental illness*												
Substance use	.						.					
No	60.6	43/71	82.4	84/102	1.00		84.6	11/13	81.1	120/148	1.00	
Yes	39.4	28/71	17.6	18/102	3.21 (1.51-6.82)	0.003	15.4	2/13	18.9	28/148	0.74 (0.15-3.59)	0.706
Depression	.						.					
No	52.1	37/71	68.6	70/102	1.00		46.2	6/13	67.6	100/148	1.00	
Yes	47.9	34/71	31.4	32/102	2.11 (1.10-4.04)	0.024	53.8	7/13	32.4	48/148	2.21 (0.70-7.04)	0.179
Anxiety	.						.					
No	76.1	54/71	86.3	88/102	1.00		84.6	11/13	85.8	127/148	1.00	
Yes	23.9	17/71	13.7	14/102	2.12 (0.94-4.79)	0.071	15.4	2/13	14.2	21/148	1.05 (0.21-5.25)	0.950
Any mental illness	.						.					
No	32.4	23/71	52.9	54/102	1.00		38.5	5/13	58.1	86/148	1.00	
Yes	67.6	48/71	47.1	48/102	2.43 (1.26-4.69)	0.008	61.5	8/13	41.9	62/148	2.09 (0.64-6.83)	0.221
*Socio-demographic factors*												
Socio-economic status+												
High	16.9	12/71	29.0	29/100	1.00		15.4	2/13	25.3	37/146	1.00	
Middle	29.6	21/71	36.0	36/100	1.74 (0.72-4.22)	0.222	15.4	2/13	28.1	41/146	1.07 (0.14-8.09)	0.951
Low	53.5	38/71	35.0	35/100	3.77 (1.58-9.00)	0.003	69.2	9/13	46.6	68/146	4.75 (0.93-24.34)	0.062
Marital status	.						.					
Married, de facto	16.9	12/71	34.3	35/102	1.00		7.7	1/13	47.3	70/148	1.00	
Divorced, separated	19.7	14/71	3.9	4/102	9.07 (2.45-33.62)	0.001	23.1	3/13	2.7	4/148	35.89 (3.11-414.73)	0.004
Never married	63.4	45/71	61.8	63/102	4.97 (1.98-12.47)	0.001	69.2	9/13	50.0	74/148	22.68 (2.44-210.95)	0.006

OR = 1.00 (Referent group).

*‘n’ refers to number of cases/controls in each exposure category. ‘N’ refers to total number of cases/controls.

+ Socio-economic status based on composite measure of household income and educational achievement.

**Table 2:** Relation of suicide to mental disorders and socio-economic status in young adults aged 18–34 years, odds ratio (OR) from multivariate models (2003–2008, New South Wales, Australia)

	**OR (95%CI) (a)**	**P value**	**OR (95%CI) (b)**	**P value**	**OR (95%CI) (c)**	**P value**
Males						
Substance use disorder						
No	1.00		1.00		1.00	
Yes	3.58 (1.60-7.99)	0.002	3.26 (1.45-7.36)	0.004	2.62 (1.11-6.15)	0.027
Affective disorder						
No	1.00		1.00		1.00	
Yes	3.08 (1.31-7.26)	0.010	3.63 (1.48-8.91)	0.005	2.80 (1.10-7.14)	0.030
Anxiety disorder						
No	1.00		1.00		1.00	
Yes	5.91 (1.38-25.26)	0.017	5.25 (1.18-23.45)	0.030	4.13 (0.85-20.22)	0.080
Socio-economic status						
High	1.00				1.00	
Mid	1.58 (0.61-4.09)	0.350			1.62 (0.58-4.53)	0.357
Low	3.08 (1.21-7.82)	0.018			2.92 (1.04-8.16)	0.042
*Linear trend*	*1.77 (1.12-2.81)*	*0.015*			*1.72 (1.03-2.85)*	*0.037*
Females						
Substance use disorder						
No	1.00		1.00		1.00	
Yes	2.24 (0.51-9.95)	0.287	2.57 (1.39-4.78)	0.003	1.12 (0.17-7.58)	0.905
Affective disorder						
No	1.00		1.00		1.00	
Yes	4.41 (0.95-20.56)	0.059	5.14 (2.56-10.30)	<0.001	1.28 (0.15-11.08)	0.825
Anxiety disorder						
No	1.00		1.00		1.00	
Yes	33.67 (4.29-264.17)	0.001	67.93 (14.03-328.79)	<0.001	43.55 (2.56-741.34)	0.009
Socio-economic status+						
High	1.00				1.00	
Mid	1.52 (0.17-13.61)	0.706			0.88 (0.08-10.00)	0.918
Low	4.78 (0.73-31.35)	0.103			5.18 (0.68-39.59)	0.113
*Linear trend*	*2.29 (1.10-5.87)*	*0.082*			*2.59 (0.92-7.33)*	*0.073*

OR = 1.00 (Referent group).

(a) Adjusted for marital status + family history.

(b) Adjusted for marital status + family history + socio-economic status.

(c) Adjusted for marital status + family history + socio-economic status + mental disorders.

+ Socio-economic status based on composite measure of household income and educational achievement.

Associations between socio-economic status and suicide were smaller in magnitude than mental disorders for both males and females in univariate analyses (Table 1), and were attenuated following adjustment for marital status and family history of mental disorder (Table 2). For males, suicide risk was highest in the lowest socio-economic status group (OR = 3.08, 95%CI 1.21-7.82, *P* = 0.018), followed by the middle socio-economic status group (OR = 1.58, 95%CI 0.61-4.09, *P* = 0.350) compared to the highest socio-economic status group (OR = 1.00), in models not adjusting for intermediary mental disorder variables (*P* for linear trend = 0.015) (Table 2). A similar pattern was evident for females, with highest risk of suicide in those from the lowest socio-economic status (OR = 4.78, 95%CI 0.73-31.35, *P* = 0.103) and middle socio-economic status groups (OR = 1.52, 95%CI 0.17-13.61, *P* = 0.706) compared to the highest socio-economic status group (*P* for linear trend = 0.082) (Table 2).

PAF% estimates were highest for Substance Use Disorder (27.9% 95%CI 11.5%-41.2%) and Affective Disorder (25.4% 95%CI 11.0%-37.4%) in males, and Anxiety Disorders (30.1% 95%CI 28.0%-32.0%) for females (Table 3). The combined PAF% for substance use, affective, and anxiety disorders was similar in magnitude to socio-economic status for males (48.4% 95%CI, 33.3-60.1% *vs*. 46.1%, 95%CI 3.8%-69.8%) and for females (52.3% 95%CI, 37.9%-64.2% *vs*. 58.1%, 95%CI −29.1%-86.4%) (combined mental disorder estimates not shown in table).

**Table 3:** Population Attributable Fraction (PAF%) for mental disorders and socio-economic status associated with suicide (young adults aged 18–34 years), 2003–2008, New South Wales (Australia)

	**% controls exposed**	**Odds ratio (95%CI)**	**PAF% (95%CI)**
Males			
Substance use disorder			
No	82.4	1.00	
Yes	17.6	2.62 (1.11-6.15)	27.9 (11.5-41.2)
Affective disorder			
No	88.2	1.00	
Yes	11.8	2.80 (1.10-7.14)	25.4 (11.0-37.4)
Anxiety disorder			
No	97.1	1.00	
Yes	2.9	4.13 (0.85-20.22)	11.7 (5.6-17.5)
Socio-economic status			
High	29.0	1.00	
Mid	36.0	1.58 (0.61-4.09)	
Low	35.0	3.08 (1.21-7.82)	46.1 (3.8-69.8)
Females			
Substance use disorder			
No	90.5	1.00	
Yes	9.5	1.12 (0.17-7.58)	3.4 (−66.0-43.8)
Affective disorder			
No	89.2	1.00	
Yes	10.8	1.28 (0.15-11.08)	8.3 (−86.7-55.0)
Anxiety disorder			
No	95.9	1.00	
Yes	4.1	43.55 (2.56-741.34)	30.1 (28.0-32.0)
Socio-economic status			
High	25.3	1.00	
Mid	28.1	0.88 (0.08-10.00)	
Low	46.6	5.18 (0.68-39.59)	58.1 (−29.1-86.4)

OR = 1.00 (Referent group).

## Discussion

This study investigated the relative contributions to suicide risk from lower prevalence psychiatric disorders and higher prevalence low socio-economic status in a population-based case–control study of young adults in New South Wales (Australia). The higher population prevalence of lower socio-economic status compared to psychiatric disorders (estimated from the control group in the present study) resulted in similar population attributable risk estimates for socio-economic status (46.1% for males and 58.1% for females) compared to psychiatric disorders (48.4% for males and 52.3% for females) Population attributable risk estimates varied across substance use, affective, and anxiety disorders - ranging from 11.7% to 27.9% for males, and 3.4% to 30% for females.

There are a number of methodological considerations in interpreting the findings from this study. This study was designed as a population-based study of suicide for young adults in geographic catchment areas in metropolitan Sydney and the Newcastle and Hunter regions. All suicide cases in the geographic catchments were enumerated for the study period, however, ethical approval for this study required that the initial approach to the next-of-kin of decedents was made by Coronial staff based on ‘closed’ cases of suicide, and next-of-kin could indicate at this stage that they did not wish to be contacted further. As a result the cases used in the present study represent 38% of suicides in the geographic catchment for the period based on Coronial records, and 70% of those next-of-kin of suicide cases who were contacted. It is possible that those next-of-kin who consented to participate in the study were different from those who chose not to participate. The probable effects on OR estimates from selection bias are difficult to judge.

An additional source of possible selection bias relates to the enumeration of controls for this study. The control group for a population-based case–control study should represent the at-risk population from which cases would be enumerated. The sampling frame for the present study was carefully designed in consultation with the ABS (as described above) to ensure a representative population-catchment from which 18–34 year old participants could be enumerated. A comparison with census characteristics of sex and age-distributions suggests that the control group is reasonably consistent with the sex and age-distributions from the Australian census for an equivalent period in these population catchments (as described above), and is unlikely to be a major source of selection bias. A higher proportion of females were recruited in those aged 20–24 years, and a lower proportion of males were recruited in those aged 30–34 years than reported in the Australian Census data, however, age matching prevented confounding by age.

Prevalence estimates of mental disorders in the control group differed from nationally representative prevalence estimates, most likely as a result of ascertainment bias in the third-person interviews. For both males and females, substance use disorders (12.8% *vs*. 5.1%) and affective disorders (11.2% *vs.* 6.2%) in the present study were higher than those found for the general population, and for anxiety disorders (3.6% *vs*. 14.4%) were lower than the general population [11]. The effects of this source of bias on underlying prevalence estimates of mental disorders is that PAF% estimates will be over-estimated in both males and females for substance use (27.8% *vs.* 8.6%) and affective disorders (22.5% *vs.* 7.7%), and under-estimated for anxiety disorders (12.2% *vs*. 24.0%).

Although the present study may be under-powered to investigate the full range of potential confounding factors, intermediaries or effect modifiers that could affect associations between key mental disorder and socio-economic status risk factors, it includes the largest number of cases of suicide for this age-group examined in comparison with population-based controls than previously reported. A case–control study of male suicide of comparable age published in Montreal in the 1990s had a similar number of suicides but fewer controls (75 cases and 75 controls) [17].

Another methodological consideration relates to the interpretation of population attributable risk [28]. Estimates of PAF assume a causal relationship between exposure and outcome. The assumption is plausible in the present instance, given the consistent and strong association shown in numerous previous studies between mental disorder and suicide, and between socio-economic status and suicide. [10] The findings of the present study are consistent with this literature, in that the lowest SES category, in males at least, was significantly and positively associated with suicide risk.

An additional assumption of PAFs is that eliminating the exposure will not affect other risk factors. This assumption is more difficult to sustain, given the inter-relationship between social factors and mental disorders associated with suicide. However, the present analysis adjusted for SES as a common cause of both mental disorder and suicide, and also based PAF estimates for SES on estimates not adjusting for mental disorder (given the likely intermediary effect). Estimates of SES and suicide have not been over-adjusted by assuming that the ‘independent’ effect of SES can be obtained by adjusting for intermediary mental disorders, or biased due to conditioning on the common effect of both SES and unmeasured confounders [29].

A final consideration relates to the sensitivity of PAF to the underlying population prevalence estimate used. In the present study the prevalence of mental disorder or lower SES is derived from the control group. The 12-month prevalences of the selected ICD mental disorders in this study are consistent with the 12-month prevalence from normative samples [11]. For SES, the proportion of controls across SES groups reflect what would be expected in the general population for males, with approximately one third of the population in each of the low, middle and high SES groups. However, there was a higher proportion of women in lower SES groups (47%) and lower proportion in higher SES groups (25%) than would be expected in the general population.

Despite the methodological limitations noted above, the findings in the present study are generally consistent with previous studies of attempted suicide in Australia [14,15], and also with findings of a recent systematic review of population-based individual-level studies of attributable risk associated with socio-economic status measures, ICD/DSM mental disorders and suicide [10]. These studies show that socio-economic status, particularly the modifiable SES measure of educational achievement, have a similar magnitude of attributable risk as common mental disorders.

## Conclusion

These findings imply that prevention initiatives that seek to modify the distribution of socio-economic deprivation in a population would have similar effects as initiatives that focus on reducing the prevalence of mental disorders. The reduction of relative socio-economic disadvantage, particularly relating to educational achievement, is also a potential proxy for other specific antecedent exposures that might be reduced and represents a specific target for prevention that could be made more prominent in national policies and strategic plans relating to suicide and mental health.

It is likely that mental disorders are intermediaries between socio-economic status and suicide (although prior mental health status may affect socio-economic circumstances), and thus interventions focussing on more distal ‘lower risk’ social determinants are likely to have an effect on mental health outcomes, in addition to suicidal behaviour. Population-based approaches targeting social deprivation have been proposed [30], particularly relating to inter-generational social deprivation [31] the success of which requires lead-times of years (if intervening in childhood). However, more immediate interventions targeting socio-economic inequality and social mobility by focusing on income-level interventions that move families out of poverty have also been associated with improvements in mental health [32,33].

## Competing interests

The authors declare that they have no competing interests.

## Authors’ contributions

AP contributed to the conception and design of the study, the collection of data, and analysis and interpretation of data, and drafted the manuscript. SM contributed to the conception and design of the study, analysis and interpretation of data, and critical revisions of the manuscript. CH contributed to the collection of the data, analysis and interpretation of data, and critical revisions of the manuscript. GC contributed to the conception and design of the study, and critical revisions of the manuscript. MD contributed to the conception and design of the study, and critical revisions of the manuscript. JD contributed to the conception and design of the study, and critical revisions of the manuscript. RT contributed to the conception and design of the study, analysis and interpretation of data, and critical revisions of the manuscript. All authors read and approved the final manuscript.

## Pre-publication history

The pre-publication history for this paper can be accessed here:

http://www.biomedcentral.com/1471-244X/14/68/prepub
